# Association of car ownership and physical activity across the spectrum of human development: Modeling the Epidemiologic Transition Study (METS)

**DOI:** 10.1186/s12889-015-1435-9

**Published:** 2015-02-21

**Authors:** David A Shoham, Lara R Dugas, Pascal Bovet, Terrence E Forrester, Estelle V Lambert, Jacob Plange-Rhule, Dale A Schoeller, Soren Brage, Ulf Ekelund, Ramon A Durazo-Arvizu, Richard S Cooper, Amy Luke

**Affiliations:** Stritch School of Medicine, Loyola University Chicago, Maywood, IL USA; Institute of Social & Preventive Medicine, Lausanne University Hospital, Lausanne, Switzerland; Unit for the Prevention and Control of Cardiovascular Disease, Ministry of Health, Victoria, Mahe Seychelles; Tropical Medicine Research Institute, University of the West Indies, Mona, Kingston Jamaica; Research Unit for Exercise Science and Sports Medicine, University of Cape Town, Cape Town, South Africa; Kwame Nkrumah University of Science and Technology, Kumasi, Ghana; University of Wisconsin, Madison, WI USA; MRC Epidemiology Unit, Addenbrooke’s Hospital, Cambridge, UK

**Keywords:** Physical Activity, Socioeconomic Status, African American, African Populations, Automobile Ownership

## Abstract

**Background:**

Variations in physical activity (PA) across nations may be driven by socioeconomic position. As national incomes increase, car ownership becomes within reach of more individuals. This report characterizes associations between car ownership and PA in African-origin populations across 5 sites at different levels of economic development and with different transportation infrastructures: US, Seychelles, Jamaica, South Africa, and Ghana.

**Methods:**

Twenty-five hundred adults, ages 25–45, were enrolled in the study. A total of 2,101 subjects had valid accelerometer-based PA measures (reported as average daily duration of moderate to vigorous PA, MVPA) and complete socioeconomic information. Our primary exposure of interest was whether the household owned a car. We adjusted for socioeconomic position using household income and ownership of common goods.

**Results:**

Overall, PA levels did not vary largely between sites, with highest levels in South Africa, lowest in the US. Across all sites, greater PA was consistently associated with male gender, fewer years of education, manual occupations, lower income, and owning fewer material goods. We found heterogeneity across sites in car ownership: after adjustment for confounders, car owners in the US had 24.3 fewer minutes of MVPA compared to non-car owners in the US (20.7 vs. 45.1 minutes/day of MVPA); in the non-US sites, car-owners had an average of 9.7 fewer minutes of MVPA than non-car owners (24.9 vs. 34.6 minutes/day of MVPA).

**Conclusions:**

PA levels are similar across all study sites except Jamaica, despite very different levels of socioeconomic development. Not owning a car in the US is associated with especially high levels of MVPA. As car ownership becomes prevalent in the developing world, strategies to promote alternative forms of active transit may become important.

## Background

Physical activity (PA) is assumed to be lower in developed than in non-developed nations, which is thought to contribute to the high rates of obesity observed in the former [1], although empirical evidence is inconsistent [2]. Reduced PA has been hypothesized to result from technological advances associated with economic development in transport, labor, housekeeping, meal preparation and leisure-time pursuits, although socioeconomic development may also provide opportunities for increasing leisure time physical activity in middle [3] and high income countries such as the Netherlands [4,5]. The types of occupations that predominate in developed nations tend to be non-manual or service occupations with low PA requirements, whereas populations in developing nations are more likely to be engaged in manual occupations requiring greater PA [6]. For example, 56% of the population of Ghana is engaged in agriculture, vs. under 1% in the United States [7]. All things being equal, common-sense dictates that individuals living in developed nations should have lower energy requirements than those living in non-developed nations; however, this claim has not been substantiated by prior research [8].

Alternatively, several compensatory mechanisms may increase PA in developed nations. Labor-saving tools may increase productivity without reducing PA [9], while greater leisure time may increase opportunities for PA in the form of exercise [10]. PA may also be reduced in developing nations, if workers engaged in agriculture, construction, and other manual occupations do not engage in leisure-time physical activity, spend less time in active commuting (walking, bicycling) to and from work, or otherwise reduce workload due to high ambient air temperature. While the opposite could also be true (e.g., people actively commute longer distances in the developing world due to poor transportation infrastructure), there is very little global research on physical activity. This is especially true for car ownership, which has been primarily researched in high-income contexts, but apart from a handful of studies, rarely in the developing world [11,12].

We report here socioeconomic findings for the 5 international African-origin cohorts that have been recruited for the Modeling the Epidemiologic Transition Study (METS) [13]. Our aim was to quantify levels of moderate to vigorous physical activity (MVPA) associated with car ownership, adjusting for socioeconomic position (SEP) using measures of income and wealth (estimated by ownership of common household items). Our focus on MVPA was motivated by its importance in preventing common chronic diseases such as cardiovascular disease and type 2 diabetes mellitus [14].

## Methods

### Sampling design and participant recruitment

Twenty-five hundred adults, ages 25–45, were enrolled in METS between January 2010 and December 2011, as described in detail elsewhere [13]. In brief; five hundred participants, 46% male, were recruited in each of five study sites: rural Ghana, a peri-urban South Africa township, mixed urban/rural Seychelles (island of Mahé), urban Jamaica and suburban Chicago. As described in our protocol paper [13], the five sites had the following characteristics:

- **Ghana:** The in-country study sites include the town of Nkwantakese in the Afigya-Kwabre District of the Ashanti Region of Ghana and its surrounding villages. The town is situated to the southwest of Agona Ashanti the District Capital, and is about 20 km from Kumasi with a population of approximately 17,000.
- **South Africa:** Khayelitsha is the 3rd largest township in South Africa and is adjacent to the city of Cape Town. The population is about 500,000 with 80 percent of the residents living in temporary housing and 40 percent unemployed.
- **Jamaica:** The study site in Jamaica is in Kingston, the capital and largest city with a population of 651,880.
- **Seychelles:** In Seychelles, individuals have been recruited from the main island of the archipelago, Mahé, which includes approximately 75,000 inhabitants for a surface of 155 km2. Mahé can be qualified as semi-urban and its economy is mainly driven by tourism, industrial fishing and services. Seychelles is located approximately 1,600 km east of Kenya in the Indian Ocean, and approximately 2,000 km north of the island of Mauritius, and has a total population of about 87,000.
- **USA (Maywood):** Maywood is an African-American working class community adjacent to the western border of Chicago, Illinois, with a population of approximately 24,903 people.

All participants were of predominantly African descent. Exclusion criteria included infectious diseases, pregnancy or lactation, and, conditions severely preventing normal physical activities, e.g. lower extremity disability. The study sites were selected to represent a broad range of social and economic development as defined by purchasing power parity adjusted gross national income (PPP-GNI) and the UN Human Development Index (HDI) in 2010. The PPP-GNI per capita is the sum of value added from resident producers, taxes, and primary income from abroad, divided by the midyear population of the country, and adjusted for purchasing power parity; the GNI per capita was $3340 in Ghana, $8170in Jamaica, $11640 in South Africa, $21340 in Seychelles, and $50861 in the US [15]. The HDI takes into account not only GNI, but also life expectancy and education level; Ghana is classified as a lower-middle HDI country (HDI = 0.540), South Africa as middle (0.621), Jamaica (0.727) and the Seychelles (0.799) as high, and the US as very high (0.934) [16]. Participants are meant to be characteristic of broad differences in socioeconomic development and broad life style patterns, rather than probability samples representing the whole population; for example, 91% of all African-Americans live in urban areas [17], 87% of Seychelles residents on the island of Mahé [18], 54% of Jamaicans live in urban areas [19], 55% of black South Africans live in townships [20], and nearly half (48%) of Ghanaians live in rural areas [21].

### Ethics approval

The METS protocol was approved by the Institutional Review Board of Loyola University Chicago, IL, USA; the Health Sciences Institutional Review Board of the University of Wisconsin, Madison, WI, USA; the Research Ethics Committee of the University of Cape Town, South Africa; the Committee on Human Research Publication and Ethics of Kwame Nkrumah University of Science and Technology, Kumasi, Ghana; the Board for Ethics and Clinical Research of the University of Lausanne, Switzerland (the primary institution for the Seychelles site’s director) and the Research and Ethics Committee of Ministry of Health of Seychelles.; and the Ethics Committee of the University of the West Indies, Kingston, Jamaica. We obtained written informed consent from all study participants.

### Measurements

#### Anthropometrics and body composition

Weight (kg) and height (m) were measured to the nearest 0.1 kg and 0.1 cm respectively, on all participants in light clothing and no shoes.

#### Physical activity - accelerometer

PA was assessed using the Actical accelerometer (Phillips Respironics, Bend, OR, USA). The monitor was worn at the waist, just behind the right hip. Each participant was asked to wear the monitor at all times over 8 days (6 complete and 2 partial), including during sleep for all sites except Seychelles, where monitors were only worn during waking time; the only time the monitor should be removed was while bathing, showering, or swimming.

Accelerometry data were processed to first infer non-wear time from segments of continuous zero activity counts lasting 90 minutes or longer. Days containing 10+ hours of wear time were considered valid days and we included participants with four or more usable days. MVPA was defined using a published cut-point (1535+ counts per minute) [22,23]. We summed up all one-minute bouts of MVPA.

#### Education, wealth, class, income and occupation questionnaire

Participants completed a 54-question survey which covered general household characteristics, participant and partner’s occupation, parental education and household assets and amenities as detailed below.

##### Education Level

The number of years of education was reported by each respondent. Given that education levels have mostly local meaning within particular labor markets (sites), we created site-specific tertiles of education. We also used years of education as a continuous measure to allow comparisons across sites.

##### Individual Occupation

The occupation questionnaire was derived from the U.K. National Statistics Socio-economic Classification, 2000 edition [24]. We used the questionnaire to code for occupation and industry, then created an indicator variable for manual vs. non-manual class. Occupations coded as non-manual class included: senior, middle, and junior managers; traditional professional (e.g., scientist, physician, lawyer) and modern professional (e.g., teacher, social worker) occupations; and clerical and intermediate occupations (secretary, call center agent). Occupations coded as manual class were split into skilled manual (technical and craft, e.g., auto mechanic) and unskilled manual (semi-routine, e.g., postal worker; routine, e.g., laborer, driver, waiter; service; and farming, agriculture, and fishing occupations). Long-term unemployed (i.e., those not working in the past year) were coded as a separate occupation category.

##### Household Income

Total monthly household income was reported in local currencies. Differences in currency values for the equivalent basket of goods across sites were transformed to US equivalent purchasing power parity (PPP) based on the 2010 data for the Penn World Table [25]. PPP adjusted values for each site were: US dollar (reference: 1.00 to the US dollar); South African rand (6.07); Ghanaian cedi (0.79); Jamaican dollar (42.80); and Seychelles rupee (3.68). All household income levels are reported in PPP-adjusted US dollars and were divided by the number of household members. Due to a large number of missing values for income (n = 633), we imputed these based on available information on goods owned, occupation, age, gender, BMI, number of household members, number of household earners, participant’s education, and parental education level. Multiple imputation regression was used, with 10 imputations and negative values recoded to zero. Imputed data were more likely to have zero income (26.8%) than the non-imputed data (2.3%), which would be consistent with non-reporting due to lack of income; we assumed households were not acquiring debt, as we had no measure of debt. Finally, we defined a relative poverty measure based on work by Ruggles [26], with the cutpoint defining poverty being below half of the per capita PPP-adjusted gross national income (GNI). Although median income would be the preferred basis for a relative poverty measure, it is not available across nations.

##### Household Wealth (Household Material Goods Score)

Questions capturing household wealth were based on the Core Welfare Indicators Questionnaire from the World Bank, designed to monitor social indicators in Africa. These questionnaires have been used extensively in international research by the World Bank and United Nations to monitor demographic and socioeconomic trends in low and middle income nations, and allow for the creation of permanent income measures which reflect lifetime, rather than current, income, including future expectations of income [27]. Because consumption of goods is tied to past, current, and expected future income, housing characteristics and ownership of goods serves as a proxy for permanent income [28,29]. The proportion of participants reporting possession or access to certain goods is shown in Table 1. Both individual items and a composite score (sum) of number of goods shown in Table 2 serve as proxies for higher or lower levels of household wealth. Car ownership was considered separately from other household goods.

**Table 1:** **Goods possessed by the household (% overall and by site)**

	**Overall**	**USA**	**Seychelles**	**Jamaica**	**South Africa**	**Ghana**
Electricity	97	100	100	99	96	89
Cell phone	96	99	98	99	93	91
Television	92	100	100	98	84	81
Electric iron	90	99	100	94	90	68
Radio or CD player	88	97	97	89	72	86
Refrigerator	84	100	99	90	75	55
Watch or clock	83	99	96	88	55	78
Modern stove	81	99	98	97	83	28
DVD or VCR	80	98	95	82	65	62
Sofa	78	98	99	62	71	60
Fan	72	98	93	96	14	59
Microwave oven	52	99	49	67	36	10
Computer	43	84	61	42	9	16
Satellite Dish or Cable TV	40	83	56	48	7	4
Car or truck	36	86	37	34	12	12
Telephone land line	34	69	58	20	15	6
Bicycle	30	68	16	38	10	16
Sewing machine	29	23	43	16	26	37
Air conditioner	23	93	7	8	2	2
*Mean # Goods (SD)*	*11.3 (4.0)*	*15.9 (1.7)*	*13.0 (2.1)*	*11.7(2.8)*	*8.2 (2.8)*	*7.7 (3.2)*
*HH owns 13+ goods (%)*	*42.4*	*95.8*	*62.1*	*42.0*	*5.4*	*5.3*

note: p < 0.001 for differences across sites in mean goods owned and whether households own 13 or more goods.

**Table 2:** **Site specific demographics, all participants with valid physical activity and socioeconomic data (N = 2,101)**

	**USA**	**Seychelles**	**Jamaica**	**South Africa**	**Ghana**	**p-value across sites**
	**(n = 427)**	**(n = 415)**	**(n = 379)**	**(n = 469)**	**(n = 411)**	
PPI adjusted Gross National Income (GNI)^1^ per capita in US$	50,861	21,340	8,170	11,640	3,340	
Human Development Index (HDI), 2010	0.934	0.799	0.727	0.621	0.540	
Age (SD)	35.5 (6.3)	36 (5.6)	34.6 (6.1)	33.6 (5.8)	34.6 (6.6)	<0.001
Male sex (%)	48.0	48.7	51.2	47.8	42.3	0.15
Body mass index (kg/m^2^) (SD)	32.1 (8.7)	26.8 (5.4)	26.3 (6.3)	27.5 (8.2)	24.1 (4.4)	<0.001
Obese (BMI > =30) (%)	53.6	23.9	25.6	31.8	9.5	<0.001
Years of education (SD)	13.3 (2.2)	12.9 (2.3)	10.7 (2.0)	9.8 (2.3)	8.4 (4.0)	<0.001
Household income^1^ per person in US$ (SD^2^)	11,652 (10,700)	10,779 (7,505)	7,042 (8,761)	886 (1,251)	1,272 (6,205)	<0.001
Proportion in relative poverty^3^ (%)	89.7	59.5	49.6	98.5	85.6	<0.001
Manual laborer or farmer (%)	48.7	41.4	63.9	87.4	75.7	<0.001

^1^PPI = purchasing power parity in 2011, in US dollars per year. See http://data.worldbank.org/indicator/NY.GNP.PCAP.PP.CD for national average based on PPI.

^2^Standard deviation for imputed income based on weighted average across 10 imputed datasets; inverse variance used for weights.

^3^Proportion falling below half PPI-GNP per capita.

#### Statistical analysis

Out of 2,506 participants, we excluded 204 participants with insufficient accelerometer data. A further 201 participants were excluded due to missing socioeconomic covariates, yielding an analysis dataset of 2101 (84% of the original population). All analyses were conducted in Stata Version 12 (College Station, TX, USA). We calculated means and standard deviations for continuous measures (age, education level, social class, income, goods score, and BMI), and proportions for categorical variables (female gender, car ownership). These calculations were done by site. We also calculated mean MVPA by site for various socio-demographic characteristics. Finally, we modeled MVPA in multivariable analysis containing car ownership adjusted for site, age, gender, income, and other household goods (Model 1). A second model (Model 2) additionally adjusted Model 1 for BMI, which tends to be associated with decreased PA, and thus may mediate relationships between socioeconomic characteristics and MVPA. We compared marginal values across sites, holding covariates constant at their overall mean values using the Stata “margins” command.

##### Assessment of the Positivity Assumption

It is common in studies using socioeconomic variables to have imbalances of variables across groups, a problem known as “structural confounding” [30,31], which is a violation of the positivity assumption, i.e., that there be nonzero (positive) numbers of participants across all combinations of strata. Following Westreich and Cole [32] and Messer [30], we searched for patterns of empty cells by converting continuous variables to quantiles (cut at the median) and running cross-tabulations with the dichotomized or naturally dichotomous variables (i.e., manual worker and car ownership) by each site. This analysis revealed several empty cells when many socioeconomic variables were included; for example, there were no high-income, low-education subjects who owned a car in South Africa or Ghana. Based on rules for sufficient adjustment sets, we chose to trade off confounding and positivity by adjusting all models for age, gender, and site, but only adjusting for two indicators of socioeconomic position (income and wealth) because they are the primary determinants (“parents” in the language of directed acyclic graphs) of car ownership that are also a associated with physical activity [33]. In other words, owning a car is heavily dependent on how much income a household currently has and how much wealth they have accumulated in the past; conditioning on income and ownership of goods (a proxy for wealth) should substantially control for confounding by occupation and education.

## Results

### Demographics

Site-specific demographics are shown in Table 2. The US site had the highest GNI per capita ($48,960), the highest level of human development (HDI = 0.934), and the highest mean income in US dollar purchasing power parity per person ($11,652). We note that the per capita income for African Americans in the US was $17,880 in 2011 [34]. Relative poverty was highest in South Africa, Ghana, and the US, and lowest in Seychelles and Jamaica (p < 0.001 across sites). Ghana showed the lowest GNI per capita ($1,260) and lowest level of development (HDI = 0.540), while South Africa had the lowest mean income ($886 per person). The prevalence of obesity ranged from 53.6% in the US to 9.5% in Ghana. Mean education levels were highest in the US (13.3 years) and Seychelles (12.9 years), and lowest in Ghana (8.4 years). The proportion of the population engaged in manual labor or farming was highest in South Africa (87.4%) and lowest in Seychelles (41.4%). All differences except for gender were significant at the p < 0.001 level.

### Household goods score

The proportion of goods owned by households varied across the 5 sites, tending to follow the levels of income and HDI (Table 1). Ownership of goods was highest in the US and lowest in Ghana, following the pattern of HDI from high to low. The only goods showing near universal ownership (>90% across sites) were electricity, cell phone, and a television. Refrigerator ownership varied widely across sites, from a high of 100% in the US and 99% in Seychelles to just 55% in Ghana. Other goods were common in the US (car, air conditioner), but not in other sites. Notably, at least 12% of all households owned a car or truck in each site, while car or truck ownership was highest in the USA (86%); differences between sites in car or truck ownership, as well as for total good scores, were statistically significant (p < 0.001).

### Physical activity by socioeconomic characteristics across sites

Levels of MVPA varied across sites but did not show a directly linear pattern based on HDI (Table 3). The highest activity of moderate or higher intensity was recorded in South Africa (37.6 min/day; SD = 31.1) and Ghana (35.3 min/day; SD = 23.1), while the lowest was in the US (24.3 min/day; SD = 29.8) and Jamaica (24.6 min/day; SD = 20.7); Seychelles was intermediate. Unadjusted associations of MVPA with socioeconomic characteristics are reported in Table 3. Greater education was associated with lower physical activity only in the US (48.2 min/day for low education vs. 19.7 min/day for high; overall p for trend < 0.001), while Seychelles showed the greatest difference between middle and high education (34.5 min/day for middle education vs. 26.8 min/day for high; overall p for trend = 0.037). Likewise, unskilled manual occupations were associated with greater MVPA in the US site (30.4 min/day), but skilled manual occupations showed the highest levels of MVPA in Seychelles (37.1 min/day) and Ghana (41.8) min/day). Relative poverty (below half of the average PPI-GNI per capita) was only associated with MVPA in Seychelles, where MVPA was greater (31.6 min/day) for poverty than for non-poverty households (27.0; p for trend = 0.033). In all sites except the US and Seychelles, unemployment was associated with lower levels of MVPA. Greater number of material goods was associated with lower MVPA in three sites (US, Seychelles, and Jamaica); in addition to these sites, Ghana also showed an association of lower MVPA with car ownership. Other means of defining socioeconomic position, including site-specific educational tertiles, social class based on workplace roles [35], and the full Standard Occupational Classification [24], showed similar patterns of association with MVPA (data not shown).

**Table 3:** **Mean accelerometer measured moderate to vigorous physical activity, in minutes per day (1 minute bouts, with standard deviation) by site and socioeconomic characteristics for subjects with complete socioeconomic data (n = 2,101)**

	**USA**	**Seychelles**	**Jamaica**	**South Africa**	**Ghana**
	**(n = 427)**	**(n = 415)**	**(n = 379)**	**(n = 469)**	**(n = 411)**
Mean MVPA	24.3 (29.8)	29.7 (21.3)	24.6 (20.7)	37.6 (31.1)	35.3 (23.1)
Years of education (overall tertiles)					
Lowest tertile	48.2 (49.6)	29.9 (22.4)	27.5 (20)	37.9 (33)	36 (23.3)
Middle tertile	27.1 (33.1)	34.5 (26)	23.4 (20.1)	37.3 (29.2)	32.1 (19.7)
Upper tertile	19.7 (22.1)	26.8 (17.1)	20.5 (24.8)	<5 obs	32.4 (23.8)
*p for continuous*	<0.001*	0.037*	0.0143*	*0.508*	*0.526*
Unskilled manual, skilled manual vs. non-manual					
Non-manual occupation	16.6 (18.1)	24.9 (17.4)	20.4 (18.8)	30.2 (21.0)	29.7 (20.2)
Skilled manual	23.0 (23.7)	37.1 (23.8)	26.5 (25.0)	49.7 (33.0)	41.8 (22.8)
Unskilled manual	30.4 (35.6)	33.3 (23.5)	25.8 (20.2)	38.4 (32.0)	35.2 (23.2)
*p for difference (F-test)*	<0.001*	<0.001*	*0.089*	*0.159*	0.033*
Employment status					
Employed	26.0 (28.0)	30.4 (22.5)	26.5 (22.2)	38.5 (29.5)	35.3 (25.3)
Unemployed	32.6 (36.4)	26.9 (16.9)	18.8 (16.4)	34.0 (27.4)	22.5 (14.2)
*p for difference*	<0.001*	*0.285*	<0.001*	*0.136*	<0.001*
Relative poverty					
Poverty	23.8 (29.8)	31.6 (21.5)	23.8 (18.7)	37.4 (31)	35.8 (23)
Not poverty	28.9 (29.8)	27.0 (20.8)	25.4 (22.5)	54.5 (38.5)	32.7 (23.3)
*p for continuous*	*0.282*	*0.033*	*0.442*	*0.150*	*0.354*
Material goods scale					
0 to 11	52.5 (64.3)	32.3 (21)	27.8 (20.9)	37.7 (31.5)	35.5 (23)
12 to 18	23.7 (28.5)	28.8 (21.4)	22.1 (20.2)	37.1 (28.5)	33.8 (23.5)
*p for continuous*	<0.001*	0.001*	0.005*	*0.345*	*0.056*
Car ownership					
No	46.6 (45.4)	33.7 (20.1)	26.8 (20.7)	38.2 (31.7)	36.6 (23.2)
Yes	20.4 (24.2)	23.0 (21.8)	20.5 (20)	33.8 (26.5)	25.8 (19.3)
*p for difference*	<0.001*	<0.001*	0.005*	*0.316*	0.002*

^1^PPI = purchasing power parity in 2011, in US dollars per year, per household member.

^2^Household income categories differ across imputations, precluding use of multiple imputation (MI). Unimputed means are reported. P-values are based on continuous income in an MI model.

*denotes p < 0.05.

^3^PPI = purchasing power parity in 2011, in US dollars per year, per household member. See http://data.worldbank.org/indicator/NY.GNP.PCAP.PP.CD for national average based on PPI. See Table 2 for mean values by site.

### Adjusted measures of association between socioeconomic characteristics and PA

Regression analyses were used to distinguish independent relationships between socioeconomic characteristics and BMI and relevant exposure variables, controlling for site (Table 4). The first column (labeled “Univariate”) reports unadjusted (crude) associations of MVPA with car ownership, site, age, gender, and socioeconomic characteristics included in the models (material goods score and income). Each additional good owned was associated with 0.47 fewer minutes of MVPA (SE = 0.22) once household car ownership, income, age, gender, and site were taken into account (Table 4, Model 1); on the other hand, owning a car was associated with 12.6 fewer minutes of MVPA (SE = 1.4). These results were robust to restricting the definition of car ownership to those who had their own personal care that was not shared with other household members; defining as personal car ownership was associated with 16.1 minutes fewer MVPA (p < 0.001). We note that adjustment for material goods score and car ownership (Table 4, Model 2) eliminated mean differences in MVPA across four of the five sites (Jamaica being the exception). Adjustment for BMI did not modify these relationships (Model 2), except to amplify differences between sites. Neither TV nor bicycle ownership were associated with MVPA (data not shown). With the exception of car ownership, there is no support for associations between SEP and MVPA differing by site.

**Table 4:** **Linear regression of minutes of moderate to vigorous physical activity across sites in 1 minute bouts (standard error in parentheses)**

**MVPA**	**Univariate**	**Model 1**	**Model 2**
		**(Car + material goods)**	**(BMI + model 1)**
Site (vs. USA)			
Seychelles	5.5 (1.8)*	−1.8 (1.8)	−4.3 (1.8)*
Jamaica	0.4 (1.8)	−8.3 (1.9)*	−10.7 (1.9)*
South Africa	13.4 (1.7)*	1.3 (2.3)	−0.22 (2.3)
Ghana	11.0 (1.8)*	0.0 (2.3)	−3.5 (2.4)
Female (vs. male)	−19.0 (1.1)*	−20.6 (1.0)*	−18.0 (1.1)*
Age (per year)	−0.1 (0.09)	−0.14 (0.08)	0.00(0.08)
Income per person ($10 k)	−2.6 (0.69)*	0.52 (0.78)	0.52 (0.77)
# Goods owned	−1.3 (0.15)*	−0.47 (0.22)*	−0.36 (0.22)
Car owned (vs. no)	−12.0 (1.2)*	−12.6 (1.4)*	−12.5 (1.4)*
BMI (per kg/m^2^)	−1.02 (0.08)*		−0.53 (0.08)*
Constant	24.3 (1.3)	77.7 (5.0)	87.0 (5.1)

*denotes p < 0.05.

Note: Univariate models do not adjust for any other variables in the model. Model 1 adjusts for all variables in the table except for body mass index (BMI), including site indicator variable, gender, age, income, number of goods owned, and car ownership; model 2 adds BMI to these variables in model 1.

Includes subjects with complete covariate information (n = 2,101).

### Car ownership by site

The association between household car ownership and MVPA differed across sites (Table 5). Upon adjustment for confounders, car owners in the US had 24.3 (SE = 3.2) fewer minutes of MVPA per day, compared to those who did not own a car. In the other sites, car ownership was associated with more modest differences in MVPA, ranging from 5.6 (3.3) fewer minutes in South Africa to 12.1 (2.4) fewer minutes in Seychelles. Furthermore, participants who did not own a car in non-US sites all showed significantly lower levels of MVPA than comparable participants in the US. Using marginal effects at the mean values across sites, car owners show similarly low levels of MVPA whether they lived in the US (marginal level of 20.7 min/day, holding all other values constant at the mean value) or in one of the other four sites (24.9 min/day). On the other hand, non-car owners in the US showed much higher levels of MVPA (45.1 min/day) than in the non-US sites (34.6 min/day). These results were robust to the differing definition of personal car ownership (as opposed to household ownership) across sites; for example, the original car ownership variable was associated with 24.3 (SE = 3.2) fewer minutes MVPA in the US site, while personal car ownership was associated with 23.8 (SE = 2.6) fewer minutes MVPA.

**Table 5:** **Linear regression of minutes of moderate to vigorous physical activity (1 minute bouts), by car ownership and site (SE in parentheses) (n = 2101)**

	**No interactions** ^**1,2**^	**No confounders**	**Adj. confounders** ^**2**^	**Confounders** ^**2**^ **+ BMI**
No car				
US	*ref.*	*ref.*	*ref.*	*ref.*
Seychelles	−1.8 (1.8)	−13.6 (2.6)*	−12.2 (3.3)*	−14.1 (3.3)*
Jamaica	−8.3 (1.9)*	−19.1 (2.7)*	−19.3 (3.4)*	−21.2 (3.3)*
S. Africa	1.3 (2.3)	−8.8 (2.6)*	−9.9 (3.5)*	−10.9 (3.4)*
Ghana	0.0 (2.3)	−13.4 (2.6)*	−10.4 (3.5)*	−13.5 (3.5)*
Car				
USA	−12.6 (1.4)*	−26.1 (3.5)*	−24.3 (3.2)*	−23.7 (−3.2)*
Seychelles		−10.7 (2.6)*	−12.1 (2.4)*	−12.2 (−2.4)*
Jamaica		−6.3 (2.7)*	−9.9 (2.6)*	−10.1 (−2.6)*
S. Africa		−4.3 (3.5)	−5.6 (3.3)	−5.7 (−3.3)
Ghana		−10.8 (3.8)*	−11.8 (3.6)*	−11.3 (−3.6)*

Note: All site*car interactions are p = 0.001 or less vs. the US.

^*1*^ *Same as Model 1, Table* 4 *.*

^*2*^ *Adjusted for age, gender, income, and material goods score.*

*denotes p < 0.05.

## Discussion

We have reported a wide range of socioeconomic correlates with MVPA across 5 sites that are vastly different in terms of economic development (by both GNI and HDI) and individual income. Adjusting for socioeconomic variables (including number of goods and car ownership) attenuated differences between sites, with the exception of Jamaica. Notably, car owners show similarly low levels of MVPA whether they lived in the US or in one of the other four sites, while non-car owners in the US showed the highest levels of MVPA.

We know of only three other studies that looked internationally across very different levels of socioeconomic development; both studies used self-reported physical activity, and only the INTERHEART (Held and colleagues) and PURE studies (Lear and colleagues) explicitly examined car ownership [11,12,36]. While the World Health Survey (Bosdriesz and colleagues) and PURE studies were population-based [11,36], the study population used by Held and colleagues consisted entirely of myocardial infarction patients [12]; none of these studies used objectively measured PA. Nevertheless, these three studies’ findings are consistent with ours, which demonstrates that socioeconomic position and car ownership are associated with lower levels of PA. Rather than attenuating differences in MVPA between sites, car ownership shows great heterogeneity between the US and other sites. In fact, participants in the US who live in a household that do not own a car have the highest levels of MVPA of any of our study subjects (approximately 45 minutes per day on average). On the other hand, study participants in Ghana, Jamaica, Seychelles, and the US whose households do own a car all showed MVPA levels of approximately 20–25 minutes per day. Part of this may reflect relative wealth associated with car ownership, particularly in low resource environments such as Ghana.

Studies of PA conducted only in the US may miss important population-level drivers of activity because there is not enough variation in lifestyle or social and physical environment [37]. Looking across international contexts provides the variance necessary to detect associations that may not be observable within-site. Car ownership may be especially salient in the US site, which is an inner suburb (Maywood, IL) with lower coverage of public transportation than the central city (Chicago). The mismatch between socioeconomic position and the built environment may underlie key health disparities [38], and these analyses do not suggest that lacking a car is overall beneficial to health in the US or any other site.

Nevertheless, the well-documented negative health effects of material disadvantage in the US reflected by not owning a car may be buffered by higher levels of MVPA. The potential protective role of MVPA in economic hardship is supported by the natural experiment of Cuba’s “special period” in the 1990s; with the end of Soviet subsidies, Cuba entered an economic crisis marked in part by extreme fuel scarcity. During this time, walking and bicycling became the only forms of transit available to most Cubans, while the food supply also shrank; this was followed by declining obesity prevalence and diabetes admissions [39]. Although there is some indication that MVPA has returned to pre-crisis levels, this natural experiment highlights the role of modern transportation in population-wide patterns of MVPA [40].

Our study had several strengths. This was a large-scale international comparison. We used objectively measured MVPA collected with accelerometers; studies employing only questionnaires may be prone to bias [41]. We furthermore had substantial overlap of socioeconomic characteristics by site, at least at the median; the positivity assumption over sites is met for all variables but education, and “structural confounding” is unlikely to explain associations [31]. In other words, each site shows a full range of socioeconomic measures.

We also note limitations of our study. Results presented here are cross-sectional; therefore causality cannot be determined. It is likely that substantial residual confounding remains for socioeconomic characteristics across sites, so the direct effect of site on MVPA just means that the measured variables do not fully account for differences between sites, and there is likely more (and more complexity) at work here. Complex systems approaches such as agent based models of changes to the built environment may be required for a more complete understanding of the mechanisms linking SEP and MVPA [42,43].

## Conclusion

In conclusion, socioeconomic characteristics are associated with objectively-measured MVPA level. This was especially true of car ownership. As car ownership increases across the developing world, extra efforts may be needed to ensure adequate levels of MVPA, for example, by building infrastructure that also supports active commuting [4,5]. In the US, where traveling by car has become the primary mode of transportation, the built environment has evolved such that walking, bicycling, and other forms of active transit are discouraged or even hazardous [44]. Nevertheless, such development is not inevitable as observed in European countries [5], and in developing nations such as the Agita movement in Brazil [3] and Muevete in Columbia [45].
